# Book Review: Embodiment, Enaction, and Culture: Investigating the Constitution of the Shared World

**DOI:** 10.3389/fpsyg.2019.01853

**Published:** 2019-08-13

**Authors:** Tom Froese

**Affiliations:** ^1^Institute for Applied Mathematics and Systems Research (IIMAS), National Autonomous University of Mexico (UNAM), Mexico City, Mexico; ^2^Center for the Sciences of Complexity (C3), National Autonomous University of Mexico (UNAM), Mexico City, Mexico

**Keywords:** enactive cognitive science, philosophy of mind, embodied cognition, phenomenology, sociology, anthropology, social cognition, social perception

This MIT Press volume edited by Durt et al. (2017) is concerned with investigating how people bring about a shared sociocultural world through participatory and broader collective sense-making processes, while at the same time highlighting how the participants in these social processes are themselves transformed by the world they help to bring forth. The key insight that runs through this interdisciplinary collection of 20 chapters is the irreducible nature of this interdependence between individual and collective processes: participation in, and hence the cultural reproduction of, patterned practices of the social world is only realizable via a thorough transformation of individual embodied minds.

This is evidently the case for higher-level cognitive capacities of persons, such as those based on language and more complex symbolic practices (Bickhard, 2017; Hutto and Satne, 2017; Di Paolo et al., 2018). But, perhaps unexpectedly, it is also true for early developing and lower-level capacities, and this is where this volume's emphasis and novel contribution lies. There are studies of how the patterned practices we acquire during ontogeny and in adulthood shape our embodiment (Fingerhut and Heimann, 2017; Gallese, 2017), the structure of conscious experience (Durt, 2017; Ratcliffe, 2017), body memory (Fuchs, 2017), and even basic processes of affective sense-making (Montes Sánchez and Salice, 2017). Moreover, several chapters reveal how this deep cultural permeation of our embodied mind has notable consequences for how we should think about health and its disorders (Henningsen and Sattel, 2017; Kirmayer and Ramstead, 2017; Ratcliffe, 2017).

The science of social cognition has come a long way since Searle's (1990) proposal of a concept of collective intentionality that emphasized that our lived experience of a shared world could in principle be nothing but a total hallucination of a brain-in-a-vat. Today there is an increasing emphasis on the fact that interaction with others makes a difference (Froese, 2018). And yet interpretations of the role of others for individual processes span a range of possibilities, going from contextual to enabling to constitutive (De Jaegher et al., 2010). *Contextual* explanations accept that social interaction can provide more input for neural processing, or that social factors can enter covariance relationships with neural activity, such as mirror neurons (Gallese, 2017). Yet, on that view the basis of cognitive processes remains the individual's brain, thereby keeping the brain-in-a-vat scenario a live possibility. *Enabling* explanations go one step further by arguing that certain capacities or actions are only realizable because of interactions with others (Elias, 2017; Rucinska, 2017). This is most evident when considering the role of caretakers during ontogeny, but it continues to apply during adulthood (Gallagher, 2017). On this view, certain individual capacities are best conceived of as open loops that require others in order to be closed into functional units (Fuchs, 2017, 2018). This consideration gives rise to an even stronger possibility, namely that these interactions not only enable individual structures and capacities, but are actually a *constitutive* part of them (de Warren, 2017; Di Paolo and De Jaegher, 2017). According to this radical proposal, others form an irreducible part of ourselves.

Another useful way of carving up the conceptual space is by considering the status of our relations with others. A broad division can be made between authors who conceive of this as a relationship in which the other is *directly* perceived or disclosed, and those who conceive of the content of social perception as a *representation* of the other. The former approach is accepted by most enactive, ecological, and phenomenological contributions. The latter, indirect approach is the default stance of mainstream cognitive science, but in this volume is almost absent. The direct approach can be further subdivided into whether the social relationship is enabling or also constitutive of an individual's properties and capacities. Some versions of ecological psychology seem to prefer an enabling role of others (Garofoli, 2017; Montes Sánchez and Salice, 2017; Rucinska, 2017), whereas enactive and phenomenological approaches go further by assigning a constitutive role (Bickhard, 2017; Di Paolo and De Jaegher, 2017; Moran, 2017).

Among authors defending a constitutive role there is a debate about the extent to which relations with others permeate individual existence. A conservative possibility is to accept that interactions are constitutive of many aspects of existence, but that there is a minimal self which precedes those interactions (Brinck et al., 2017; Zahavi, 2017). A radical but intriguing alternative is that the individual self should not be assigned any primacy and that it instead always already coemerges in interaction with others (Ciaunica and Fotopoulou, 2017; Ratcliffe, 2017). A codependent emergence of self-and-other can be investigated in different timescales, ranging from prenatal ontogeny and development in an individual's lifetime (Ciaunica and Fotopoulou, 2017; Gallese, 2017) to the historical constitution of patterned and symbolic practices over generations reaching pack into prehistory (Fuchs, 2017; Garofoli, 2017; Hutto and Satne, 2017). Even on evolutionary timescales life always comes from life.

In summary, this volume is a fitting sequel to the foundational MIT Press volume on enaction edited by Stewart et al. (2010). The aim of that first volume was to help establish the enactive approach as a new paradigm for cognitive science, and a key concern was to find a conceptual bridge between “low-level” sensorimotor activity and “high-level” human cognition (Froese, 2012). That original goal has been accomplished: mainstream cognitive science is increasingly taking into account the role of embodied interaction with the environment, while the enactive approach (broadly conceived) has been scaled up to the sociocultural environment. Consequently, as that approach has matured, it has also become much less concerned with criticizing the classical computationalist paradigm. As this volume nicely demonstrates, instead that approach is now more interested in forging ahead on its own terms. It is thereby developing an attractive vision for a modern cognitive science that places interaction, especially sociocultural interaction, center stage.

## Author Contributions

The author confirms being the sole contributor of this work and has approved it for publication.

### Conflict of Interest Statement

The author declares that the research was conducted in the absence of any commercial or financial relationships that could be construed as a potential conflict of interest.
